# A Prospective, Multinational Pharmacoepidemiological Study of Clinical Conversion to Sirolimus Immunosuppression after Renal Transplantation

**DOI:** 10.1155/2012/107180

**Published:** 2012-08-09

**Authors:** Bertram L. Kasiske, Bjorn Nashan, Maria Del Carmen Rial, Pablo Raffaele, Graeme Russ, Josep Campistol, Mark D. Pescovitz, Paul A. Keown

**Affiliations:** ^1^Division of Nephrology, Hennepin County Medical Center, Minneapolis, MN 55415, USA; ^2^University Medical Center Hamburg-Eppendorf, Hamburg, Germany; ^3^Instituto de Nefrología, Buenos Aires, Argentina; ^4^Fundación Favaloro, Buenos Aires, Argentina; ^5^The Queen Elizabeth Hospital, Adelaide, SA, Australia; ^6^Renal Transplant Unit, Hospital Clinic University of Barcelona, Barcelona, Spain; ^7^Indiana University School of Medicine, Indianapolis, IN, USA; ^8^University of British Columbia and Syreon Corporation, Vancouver, BC, Canada

## Abstract

This prospective pharmacoepidemiological study examined treatment and outcomes in patients converted to sirolimus (SRL) after renal transplantation. 484 subjects in 36 centres in 7 countries were followed for up to 5 years. Principal reasons for conversion were declining graft function (146/484, 30%) and side effects of prior therapy (144/484, 30%) and the major treatment combinations after conversion were SRL ± MMF (62%), SRL + TAC (21.5%), SRL + CSA (16.5%). The cumulative probability of biopsy-confirmed acute rejection (BCAR) was 5% (*n* = 22), death-censored graft loss 12% (*n* = 56) and death 6% (*n* = 22), and there was no significant relationship to the treatment combination employed. Median calculated creatinine clearance was 48.4 (29.3, 64.5) mL/min at conversion, rising to 54.1 (41.2, 69.0) mL/min at month 1, 55.7 (39.0, 73.0) mL/min at month 12, 58.6 (39.7, 75.2) mL/min at two years and 60.9 (36.0, 77.0) mL/min at three years post-conversion. The most common adverse events were hypertension (47%), hyperlipidemia (26%), urinary tract infections (25%), anaemia (24%) and diarrhea (14%), and cardiac events, hyperlipemia and CMV infection were more common in patients converted during the first year. SRL was most frequently combined with MMF after conversion, but principal clinical outcomes were not significantly influenced by the treatment combination employed in normal practice.

## 1. Introduction

Sirolimus is a potent m-TOR inhibitor that blocks growth factor-induced transduction signals mediating cell division in the immune response and oncogenesis [1–3]. Initial randomized clinical studies showed a 50% relative risk reduction in acute graft rejection when sirolimus was used in combination with cyclosporine (CSA) compared to CSA alone or in combination with azathioprine and helped to define the dosing requirements, pharmacological exposure, and toxicity [4, 5]. Subsequent randomized studies showed that sirolimus was also effective in combination with tacrolimus [6–8] and suggested that graft function, treatment continuation, and adverse event profiles were superior to those in subjects receiving sirolimus with CSA, perhaps reflecting an important pharmacokinetic interaction in this latter combination [6, 9, 10]. Sirolimus has since been used as primary therapy in several different treatment algorithms, including the use in combination with CNI at high or low doses, or as a replacement for CNI or antimetabolites [2, 11–20]. 

The demonstrated benefit of sirolimus as primary therapy, coupled with the potential reduction in virus infection and malignancy [21–24] and reduced lifetime treatment costs [25], has contributed to the use of this agent later after transplantation to improve immunosuppression or minimize treatment risks in subjects previously receiving other therapeutic agents [2, 26–34]. Subjects have commenced sirolimus generally between 1 month and 10 years after transplant principally for reasons of CNI toxicity, deteriorating graft function, or evidence of chronic allograft nephropathy. Pooled estimates in randomized and nonrandomized studies showed that serum creatinine improved slightly but significantly following conversion to sirolimus, while serum cholesterol rose [26]. There was no significant increase in the risk of acute graft rejection following institution of sirolimus therapy, but 17% to 28% of subjects discontinued the drug because of adverse effects principally due to oral ulcers, bad taste, anemia, proteinuria, pneumonia, or decreasing renal function [26].

Randomized trials often entail important selection which limit generalizability and may not reflect population risk or management [35, 36]. Establishing a sound methodological and statistical foundation for hypothesis-driven population-based comparative studies designed to optimize long-term therapy in chronic disease states requires precise understanding of disease epidemiology, detailed information regarding the clinical utilization of current therapies, and an accurate approximation of their quantitative benefit and detriment. The wide range of treatment combinations and results, the variability of risk and management, and the paucity of large population-based studies on the use of different agents often in complex treatment combinations confound evaluation in many long-term scenarios [37, 38]. This is particularly pertinent in the maintenance therapy of renal transplantation, where transition between treatments is common and the lack of information regarding the use and consequences of various combinations of principal immunosuppressive agents under normal clinical conditions makes it difficult to define preferred strategies for secondary therapy later in the course of renal transplantation. Understanding the principal treatment strategies in common practice, the prevalent use of sirolimus as monotherapy or in dual or triple combinations with CNI and antimetabolites and the outcomes with each of these combinations are required in order to structure effective prospective comparative trials.

We have recently reported the results of a large multinational study describing the pharmacoepidemiology, utilization, and comparative outcomes of the de novo use of these treatment combinations under conditions of normal clinical practice [39]. The current multinational prospective pharmacoepidemiological study was instituted to examine these questions when this drug was introduced as secondary therapy in the later transplant course under conditions of normal clinical practice. The objectives of the study were to document (a) the population use of sirolimus alone or in combination with other principal immunosuppressive agents, (b) the change in graft function and incidence of adverse events, and (c) the probability of biopsy-proven graft rejection, death-censored graft loss, and patient death in relation to treatment combination and timing throughout the period of observation and to establish a foundation for hypothesis-driven studies designed to optimize immunosuppressive therapy [40, 41].

## 2. Materials and Methods

### 2.1. Study Design

 This prospective, longitudinal observational study was conducted under conditions of normal clinical practice to examine treatment strategies and clinical outcomes in subjects who commenced sirolimus as conversion therapy following renal transplantation. The study employed an open cohort design [42]. Subjects were selected for transplantation and received de novo immunosuppression according to the normal clinical practice of each participating centre. They were considered eligible for entry to the current study if they were less than 75 years of age, had received a deceased or living-donor (non-HLA identical) renal transplant, did not have another transplanted organ, were able to receive oral medication, were treated with a CNI and/or purine synthesis inhibitor (MMF, azathioprine), were converted to sirolimus more than 7 days after transplantation, and were willing to provide written informed consent. To explore the influence of time of conversion, subjects were grouped for analysis purposes into two empirical, principal, rational, and approximately comparably sized exposed study cohorts in which sirolimus treatment was commenced early (<1 year) or late (>1 year) following transplantation. To explore the influence of treatment combination, subjects were grouped into three principal categories comprising treatment with SRL ± MMF, SRL + TAC, and SRL + CSA. Those in whom sirolimus was subsequently discontinued were analysed using the intent-to-treat principle. The protocol was approved by the ethics committee at each of 36 participating centres in the United States (14), Argentina (9), Brazil (5), Canada (4), Mexico (2), Belgium (1), and Australia (1). All subjects provided written informed consent. Reporting was consistent with the guidelines of the STROBE initiative [43].

### 2.2. Outcome Measures

Study data for patients converted to sirolimus were collected for the time of transplantation and discharge from hospital, for weeks 1, 4, 8, 12, 16, 20, 24, 36, and 52, and approximately every 3 months thereafter to a maximum of 5 years. These time-points were selected to parallel the routine follow-up frequency for patients in most centres participating in the study. Clinical and laboratory data from each site was reported directly from their health records by an experienced study team member. Laboratory ranges were verified for each site and updated during the study as appropriate. Acute rejection and chronic allograft injury were determined according to the Banff criteria and as employed in prior studies [39]. Graft function was measured using the Cockroft-Gault formula, and graft failure was determined as permanent loss of function, return to dialysis, or removal of the graft as reported by the site. Graft biopsy was not mandated for the study but was performed according to the normal practice of each centre. Grafts that failed were allocated an estimated creatinine clearance value of 10 mL/min up to the point of patient death or conclusion of the study. Whole blood samples were taken immediately prior to the morning dose of sirolimus or CNI, and whole blood drug levels were measured in each center by specific immunoassay or mass spectrometry. Dosing of all agents was performed based on the practice of each centre.

### 2.3. Bias and Confounding

 Formal procedures were observed to minimize potential bias and confounding. Information bias due to patient misclassification, treatment coding, or reporting error was minimized by continuous interaction with the study site, repeated cross-tabulation, and data review for verification or correction. Detection bias was minimized by the use of objective outcome measures of graft function and survival and histological confirmation of graft rejection according to the Banff criteria, and multivariate regression analysis was used to reduce protopathic bias or confounding.

### 2.4. Statistical Analysis

The primary study objective was to determine the therapeutic strategies following conversion to sirolimus. Secondary objectives included the incidence of BPAR and graft loss by treatment strategy, overall graft function, and complications. The study size was determined empirically based on the estimated rate of conversion to sirolimus, the feasible duration of study conduct, and the practical limitation of participating sites. An overall target of approximately 500 subjects recruited from over 30 sites in at least 5 countries was considered appropriate to permit a robust comparison of principal predictor and outcome variables among patients converted to sirolimus. Comparability of demographic and baseline characteristics was assessed using Fisher's exact test for categorical variables and the Wilcoxon Rank Sum test for continuous variables. The time to occurrence of principal outcomes was analysed using Kaplan-Meier survival analysis and compared using the log rank test. Missing data were not imputed, and subjects were removed from analysis at the point of loss to follow up. Grafts that failed were allocated an estimated creatinine clearance value of 10 mL/min up to the point of patient death or conclusion of the study. Cox proportional hazard models were used to measure the effect of principal recipient, graft, and treatment covariates including recipient age, gender, ethnicity, graft number, donor source, treatment prior to conversion, timing of conversion, reason for conversion, and treatment after conversion on graft outcomes and to define hazard ratios. Tests of hypotheses were two-sided unless otherwise indicated, and a *P* value of less than 0.05 was considered significant for this exploratory analysis. All analyses were conducted using SAS version 9.1.3.

## 3. Results

### 3.1. Subjects

**Figure 1:**
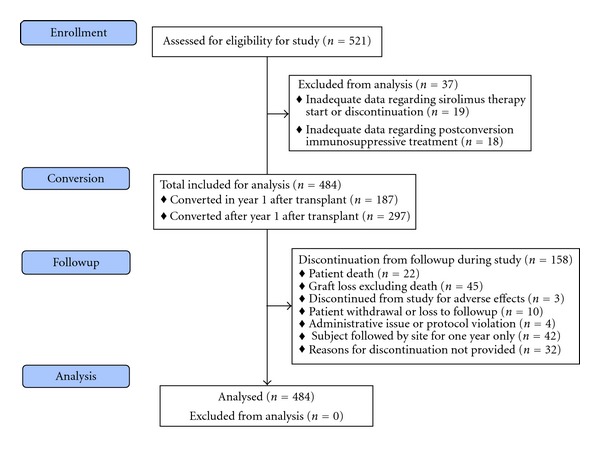
Flow diagram showing study cohort, disposition, followup, and analysis cohort.

 A total of 521 subjects were enrolled in the study, of whom 484 fulfilled the selection criteria and were included in this analysis. Their distribution and followup is shown in Figure 1. Demographic characteristics and baseline variables are shown in Table 1. Sirolimus was started between 1 and 213 months (mean 39 ± 41) after transplant, and subjects were followed for a mean of 25 ± 13 months. As shown in Table 1, declining graft function (146/484, 30%) and side effects of prior therapy (144/484, 30%) were the principal reasons for conversion to sirolimus; smaller numbers were converted as part of routine practice (40/484, 8%) because of delayed graft function (31/484, 6%), because of rejection on prior therapy (14/484, 3%), and for a variety of other reasons as shown. Approximately 40% of subjects (187/484, 39%) were converted to sirolimus within the first year after transplant, and the remainder (297/484, 61%) were converted beyond this time. Subjects converted to sirolimus within the first year were older (mean age 45 ± 14 versus 35 ± 15 years, *P* < 0.001) and more frequently Black (17% versus 10%) than those converted later in the transplant course.

### 3.2. Treatment Regimens

**Figure 2:**
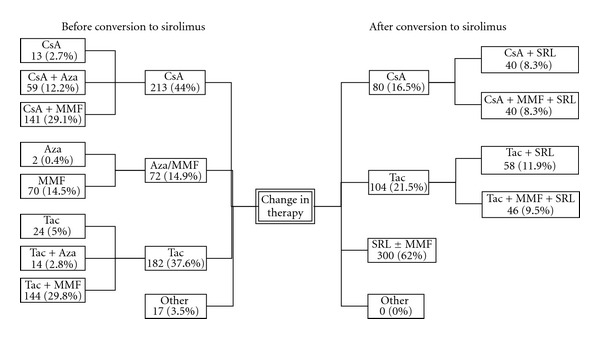
Immunosuppressive strategy before and after conversion to sirolimus (all subjects).

The principal immunosuppressive drug combinations employed before and after starting sirolimus are shown in Figure 2. A total of 207 subjects received depleting or nondepleting antibody therapy at the time of transplantation. Before starting sirolimus, 213 subjects (44%) were receiving CsA-based immunosuppression, 182 (38%) tacrolimus-based immunosuppression, 72 (15%) either azathioprine or MMF without a CNI, while 17 (4%) were receiving other agents. After conversion, 80 (17%) received sirolimus in combination with CSA and 104 (21%) with tacrolimus; 86 of these 184 subjects (47%) also received MMF. A further 300 (62%) subjects received SRL ± MMF without a CNI, of whom 58 (19%) received sirolimus alone.

**Figure 3:**
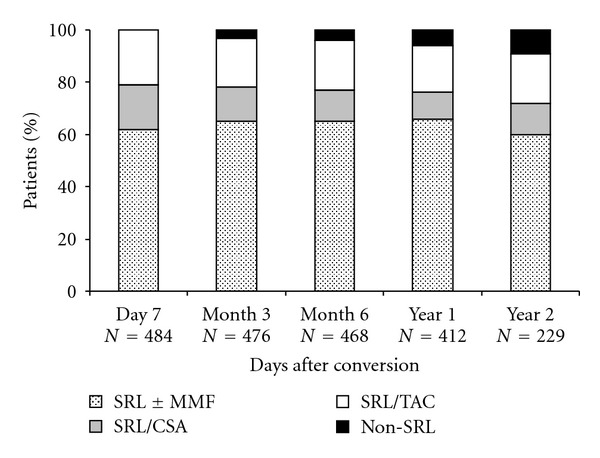
Principal immunosuppressive treatment regimens following conversion to sirolimus.

The change in principal treatment combinations after starting sirolimus is shown in Figure 3. Approximately 60% of subjects followed remained on SRL ± MMF by two years after conversion, of whom approximately 14% received sirolimus alone at this point and the remainder in combination with MMF; 19% of subjects remained on SRL + TAC and 12% on SRL + CSA. Approximately 4% of subjects received triple therapy with SRL + CSA + MMF and 7% received SRL + TAC + MMF. The treatment combination employed was not dependent on the reason for conversion. A total of 419/484 (87%) subjects who started sirolimus continued treatment throughout the following year. Reasons for discontinuation of sirolimus included side effects (25, 38%), local treatment protocol (11, 17%), and other reasons (22, 34%).

### 3.3. Immunosuppressive Dosing

The doses and blood concentrations of sirolimus, tacrolimus, and cyclosporine are shown in Table 2. The mean dose of sirolimus employed at the time of conversion was 4.3 ± 3.8 mg/day. This declined to 2.9 ± 2.1 mg/day at month 3 and 2.3 ± 1.1 mg/day at two years after conversion while the concentration rose to 10.1 ± 4.6 *μ*g/L at 3 months and declined to 8.5 ± 2.9 *μ*g/L at two years. The mean sirolimus doses employed and blood concentrations achieved were comparable between patients who were converted early or late following transplant. The mean dose of tacrolimus among patients who continued to receive this drug declined from 6.0 ± 4.1 mg/day to 4.5 ± 2.2 mg/day and that of cyclosporine from 188 ± 183 mg/day to 167 ± 107 *μ*g/L during the two years after conversion, with a commensurate decline in blood concentrations. The mean doses of both TAC and CSA were higher among those converted during the first year following transplant, consistent with the use of higher doses of these agents in the earlier posttransplant course.

### 3.4. Graft and Subject Outcomes

**Figure 4:**
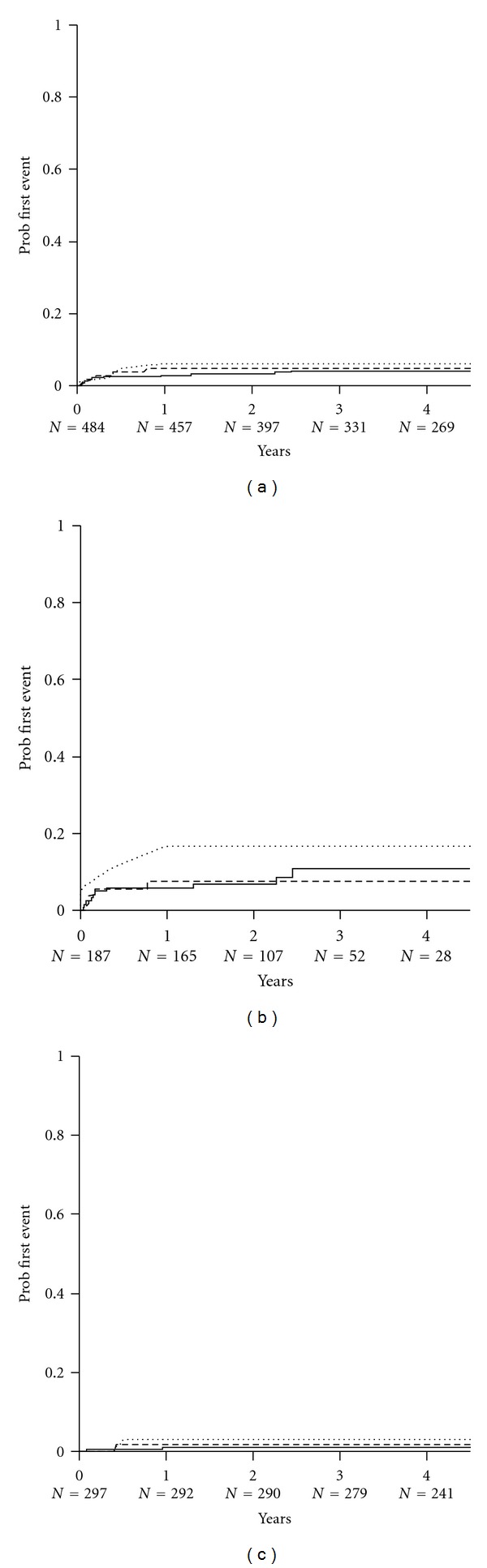
The Kaplan-Meier estimates of biopsy-confirmed acute rejection in (a) all subjects converted to sirolimus (*n* = 484), (b) subjects converted during the first posttransplant year (*n* = 187), and (c) subjects converted later after transplant (*n* = 297). Lines indicate principal treatment regimen: SRL ± MMF (solid line); SRL + CsA (dotted line); SRL + TAC (dashed line). There were no significant differences between treatment combinations.

 A total of 22 subjects (5%) experienced a biopsy-proven rejection episode following conversion to SRL. Of these, 45 (51%) were Banff grade 1a, 22 (25%) grade 1b, 15 (17%) grade 2a, 2 (6%) grade 2b, and 1 (1%) grade 3. The Kaplan-Meier (KM) estimates are shown in Figure 4. The cumulative probability of BCAR was approximately fivefold greater (10% versus 2%, *P* < 0.002) in subjects converted to sirolimus less than 1 year after transplant, consistent with the greater likelihood of rejection in the early posttransplant period. Among these subjects converted early after transplant (during the first 12 months), the cumulative probability of BCAR was higher in those converted to combination therapy with SRL + CSA (0.17; 95% CI: 0.057–0.432) and was lower in those who had commenced combination therapy with SRL + TAC (0.08; 95% CI: 0.030–0.192) although these difference in treatment regimen did not reach significance (*P* = NS). Among subjects converted later following transplant (after 1 year), the observed risk of BCAR was again higher in those receiving SRL + CSA (0.032, 95% CI: 0.008–0.123) and the lowest in those receiving SRL ± MMF (0.011, 95% CI: 0.003–0.043), although again there was no significant difference among treatment regimens (*P* = NS).

**Figure 5:**
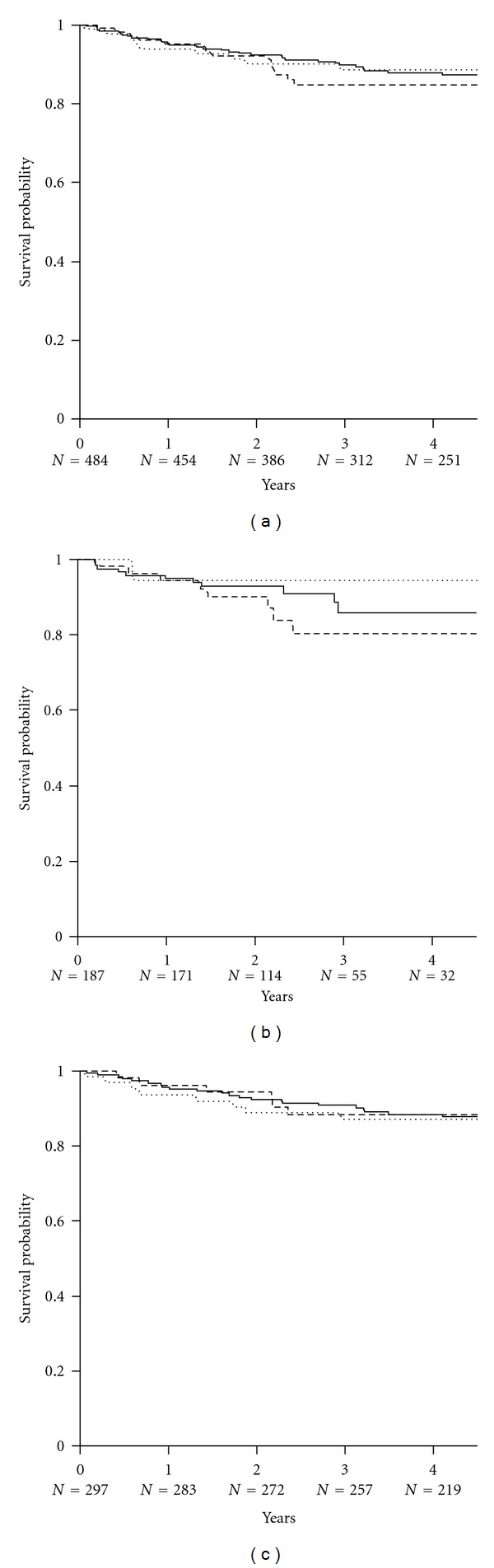
The Kaplan-Meier estimates of graft survival in (a) all subjects converted to sirolimus (*n* = 484), (b) subjects converted during the first posttransplant year (*n* = 187), and (c) subjects converted later following transplant (*n* = 297). Lines indicate principal treatment regimen: SRL ± MMF (solid line); SRL + CsA (dotted line); SRL + TAC (dashed line). There were no significant differences between treatment combinations.

A total of 56 subjects (12%) lost their graft during the study. The most frequent reasons for graft loss were chronic rejection (*N* = 31), death (*N* = 7), chronic allograft injury (*N* = 6), and acute refractory rejection (*N* = 4). The Kaplan-Meier estimates of postconversion graft survival censored for death are shown in Figure 5. The cumulative probability of graft survival by 5 years after conversion in the total study cohort was 0.87 (95% CI: 0.83–0.90), and there was no significant difference between subjects receiving SRL alone or with MMF (0.87; 95% CI: 0.82–0.91) and those receiving immunosuppressive treatment combinations of SRL + CSA (0.89; 95% CI: 0.79–0.94) or SRL + TAC (0.85; 95% CI: 0.76–0.91). No significant influences of treatment combination on graft survival were observed when subjects were considered by time of conversion. Among subjects converted early after transplant (within the first 12 months), the cumulative probability of graft survival after 2 years was 0.92 (95% CI: 0.85–0.96) in subjects converted to SLR + MMF, the predominant therapy employed, compared with 1.00 (95% CI: 0.00–1.00) in those converted to SRL + CSA and 0.92 (95% CI: 0.72–0.98) in those converted to SRL + CSA. These differences in treatment regimen did not reach statistical significance. Similarly, among subjects converted later following transplant (after 1 year), graft survival after 2 years in subjects converted to SRL alone (0.92; 95% CI: 0.80–0.97) or SRL + MMF (0.92; 95% CI: 0.86–0.96) was comparable to those converted to a combination of SRL + TAC (0.93; 95% CI: 0.77–0.98) or SRL + CSA (0.88; 95% CI: 0.71–0.95), and there was no significant difference among any of the treatment regimens.

**Figure 6:**
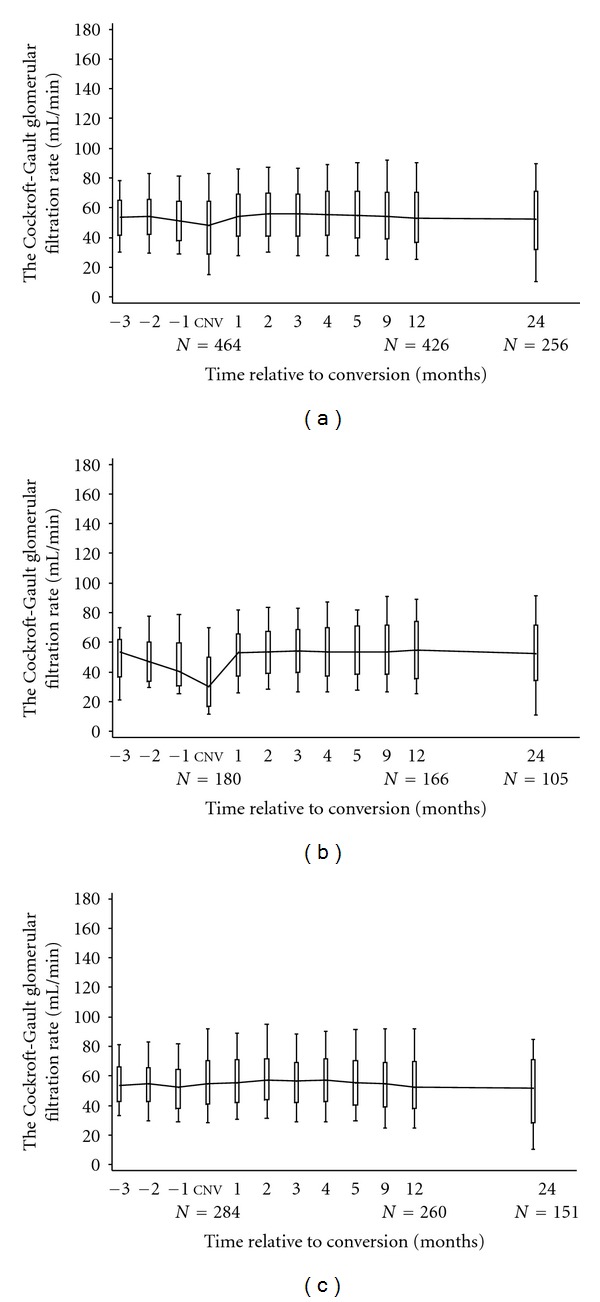
Calculated creatinine clearance before and after conversion to sirolimus in (a) all subjects converted to sirolimus (*n* = 464), (b) subjects converted during the first posttransplant year (*n* = 180), and (c) subjects converted later following transplant (*n* = 284).

Figure 6 shows graft function before and after conversion to sirolimus. The median calculated creatinine clearance for the total study cohort was 48.4 (29.1, 64.5) mL/min at the time of conversion, rising to 54.1 (41.0, 69.0) mL/min at month 1, 52.9 (36.9, 70.6) mL/min at month 12, 52.2 (32.0, 71.2) mL/min at two years, and 40.4 (10.0, 70.0) mL/min at three years after conversion. There was no significant difference in calculated creatinine clearance between subjects according to initial treatment strategy. The greatest quantitative improvement was observed among subjects converted early after transplant, where the median calculated creatinine clearance was 30.0 (17.0, 49.7) mL/min at the time of conversion, rising to 53.0 (37.6, 65.6) mL/min at month 1, 54.7 (35.5, 73.9) mL/min at month 12, 52.3 (34.2, 71.4) mL/min at two years, and 45.7 (17.0, 74.5) mL/min at three years after conversion. The change in graft function was less marked among subjects converted more than 1 year after transplant. Calculated creatinine clearance was 54.8 (41.0, 70.2) mL/min at conversion and remained stable thereafter being 55.1 (42.3, 71.2) mL/min at month 1, 52.6 (37.7, 69.5) mL/min at 12 months, 51.8 (28.3, 71.0) mL/min at two years and 34.9 (10.0, 65.9) mL/min at three years after conversion. Graft function at the time of conversion did not appear to influence the change in median calculated creatinine clearance values at one or two years following conversion in either study group (data not shown).

Adverse events are shown in Table 4. A total of 458/484 subjects (95%) reported at least one adverse event during the course of observation, of which the most common were hypertension (47%), hyperlipidemia (26%), anaemia (24%), urinary tract infections (25%), diarrhea (14%), and CMV infection (12%). Subjects converted within the first year had a lower incidence of proteinuria (5.3% versus 13.1%; *P* = 0.005), hyperuricemia (2.1% versus 7.1%; *P* = 0.019), and therapeutic agent toxicity (1.1% versus 4.7%; *P* = 0.035) but higher rates of CMV (18.7% versus 7.4%; *P* < 0.0001) and hyperlipidemia (35.3% versus 20.9%; *P* = 0.001) than subjects converted to sirolimus at a later date. Mouth ulcers, skin rash, and pneumonitis were each reported in less than 1% of subjects, while edema was more common occurring in 6–8% of patients.

**Figure 7:**
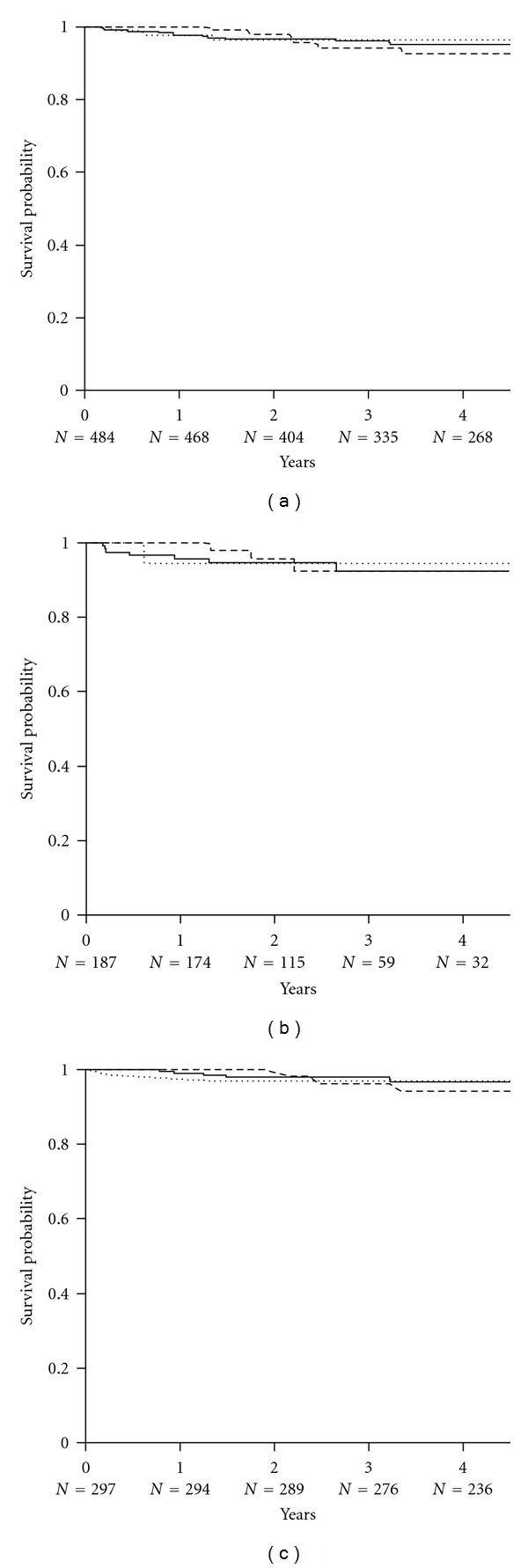
The Kaplan-Meier estimates of patient survival in (a) all subjects converted to sirolimus (*n* = 484), (b) subjects converted during the first posttransplant year (*n* = 187), and (c) subjects converted later after transplant (*n* = 297). Lines indicate principal treatment regimen: SRL ± MMF (solid line); SRL + CsA (dotted line); SRL + TAC (dashed line). There were no significant differences between treatment combinations.

By 5 years after conversion, a total of 22 subjects had died, of whom 11 (6%) had been converted within the first year and 11 (4%) beyond that point (*P* = 0.057) (Figure 7). The most common reasons for death were sepsis (*n* = 8), respiratory complications (*n* = 4), malignancies (*n* = 2, liver and gall bladder, non-Hodgkin lymphoma), myocardial infarction (*n* = 2), and 1 each from seizures, cardiac arrest, end-stage renal disease, electrolyte imbalance, diabetic ketoacidosis, or other causes.

### 3.5. Multivariate Analysis

Cox models were used to explore the relationship between time to first BCAR, graft loss, and death adjusting for principal covariates including time of conversion, recipient age, ethnicity, prior kidney transplant, PRA, induction immunosuppression (depleting, nondepleting, or none), and organ donor source (Table 3). The risk of death was significantly associated with recipient age at the time of transplant as anticipated (HR 1.069) and nonsignificantly increased in recipients of a prior kidney (HR 3.02) or deceased donor graft (HR 2.71). There was a trend towards a higher risk of graft loss in Black recipients (HR 1.63) and recipients of a prior transplant (HR 1.53) or deceased donor graft (HR 1.31). The risk of graft rejection was significantly associated with conversion in the first posttransplant year (HR 6.60) or the use of anti-CD25 antibody induction (HR 3.71), and there was a nonsignificant trend towards an increase in the risk of rejection in recipients of a deceased donor graft (HR 1.98).

## 4. Discussion

The effective use of novel therapeutic agents in the complex combinatorial treatment strategies employed in normal clinical practice, and the evaluation of risk/benefit, health impact, and costs of subject care with these treatment combinations, require detailed information that is frequently not available from randomized prospective studies. Systematic literature review and meta-analysis of existing trials [13, 14, 26, 44] and well-designed, methodologically sound pharmacoepidemiologic studies can be used to validate preliminary results using large population study methods [42, 45–47] and can provide important insights to the linkages between real-world outcomes and their multilevel determinants [48]. This study, which is the largest prospective purely observational evaluation of conversion to sirolimus under normal practice conditions, is designed to complement these data and to provide the foundation for subsequent hypothesis-driven trials of maintenance immunosuppression. 

Treatment combinations were consistent with study reports describing the growing use of sirolimus as conversion therapy since its approval in 1999 [4, 5, 8, 18, 49]. Before conversion, approximately 80% of subjects were receiving a CNI, consistent with the use of these agents reported by Kaufman et al. [49]; after conversion, approximately 40% of subjects remained on these medications and approximately 60% received CNI-free therapy. SRL + MMF was the most common therapeutic regimen after conversion, used in approximately 50% of subjects independent of the reason for conversion, while SRL + TAC and SRL + CSA with or without MMF were each employed in approximately 20% of subjects. Changes in treatment regimen occurred over time particularly in subjects converted within the first year after transplant, the proportion of subjects receiving MMF declined slightly, and there was a corresponding increase in the use of CNIs despite the growing recognition of their pharmacokinetic interaction with sirolimus and their propensity for cumulative nephrotoxicity [6, 9, 14]. The mean blood concentrations of sirolimus remained constant throughout the period of observation towards the lower margin of the target range of approximately 8–16 *μ*g/L specified in individual trials of CNI withdrawal [13] or conversion therapy [26].

Prospective randomized studies provide no consistent evidence for incremental risk of BCAR after conversion to SRL [24, 26], and nonrandomized studies suggest only a minor increment (pooled rate: 3.4%) [26], while a randomized study and meta-analysis of CNI withdrawal show an incremental risk of BCAR of approximately 1–6%, respectively [13, 24]. However, although acute graft rejection appears to be uncommon following these changes in maintenance immunosuppressive therapy, there is little information on the rejection rate according to reason for or time of conversion. The data reported here show that BCAR was more common among subjects converted to SRL in the first year (particularly within the first 2 months, rather than later after transplant (9% versus 2%), consistent with the normal timing of this event [50, 51]. There was no significant difference in rejection rates between individual treatment regimens in either subjects who were converted early or later following transplant and no indication that subjects remaining on CNI had superior outcomes to those in whom these drugs were withdrawn. There was no significant difference in BCAR according to the reason for conversion, although numbers of subjects converted for prior acute rejection were small.

Calculated creatinine clearance improved in patients converted to SRL within the first year and remained stable throughout the period of followup, consistent with previous data from both prospective randomized and nonrandomized studies [26]. Graft function remained stable in those converted later than 1 year, although no comparable increase in calculated creatinine clearance was observed. This difference may reflect a variety of physiological effects including the early use of sirolimus to avoid prolonged CNI use in subjects with delayed graft function or the potential reversibility of early CNI toxicity by comparison with later graft injury, but the exact etiology cannot be precisely determined from the data available in this study. Graft histology and function are difficult to predict following conversion to sirolimus, but current prospective studies suggest that they may improve in subjects converted from CsA while remaining comparable to those maintained on Tac [52, 53]. The probability of graft loss was slightly higher among subjects converted during the first year by comparison with those converted at a later date, but there was no apparent relationship between graft function at the time of conversion and change in function over the next two years in either group. This differs from other reports which suggest that poor graft dysfunction may be a risk factor in patients converted to sirolimus and requires more detailed investigation [24, 54–56]. The current study did not examine quantitative change in proteinuria following conversion. The mechanisms of sirolimus-associated glomerular injury are not yet fully delineated, but podocyte injury and glomerulosclerosis may be related to reduction of VEGF synthesis, AKT1 phosphorylation and expression of the transcription factor WT1 (required for maintaining podocyte integrity), the slit-diaphragm protein nephrin, and the cytoskeletal adaptor protein Nck, along with altered actin formation, leading to reduced podocyte adhesion and motility [57–59].

The adverse events reported in this population-based observational study were qualitatively comparable to those from prior randomized trials, with hypertension, hyperlipidemia, anaemia, and urinary tract infections being the most common reported complications. Surprisingly, the incidence of mouth ulcers and pneumonitis was relatively low, though the former may have been included in other GI problems reported. However, as detailed by Naesens, studies generally report a higher incidence of ulceration, edema, proteinuria, and other secondary effects when patients are converted early after transplant [53], while the majority of subjects reported here were converted after the first 3 months after transplant. While sirolimus has been associated with an increased risk of de novo diabetes [60], the rates observed here are consistent with those reported in a previous systematic review and meta-analysis, which showed a wide range in incidence and a proclivity for this complication in subjects receiving tacrolimus [61]. There was no important difference in this or other studies of the incidence of lymphocele [2, 12, 14] or malignancy according to time of conversion [1, 12, 14, 62].

The results of this large international longitudinal, pharmacoepidemiological study suggest that sirolimus provides acceptably safe and effective immunosuppression following conversion and offer important hypothesis-generating observations that may form the basis for subsequent prospective randomized studies [63, 64]. Sirolimus treatment regimens and dosing ranges in normal practice are highly heterogeneous, and dominant conversion strategies with clear superiority in terms of effectiveness or safety have not yet emerged. Although the study was not designed for rigorous statistical comparison of outcomes among these treatment regimens, there was no significant difference in primary graft or patient outcomes between subjects receiving sirolimus in combination with a CNI or those in whom CNI treatment was discontinued. However, more detailed evaluation of treatment combination, dosing, and blood concentrations is now underway to examine the impact on these principal outcomes and graft function.

While stringent efforts were made to minimize critical study biases, including selection bias, information bias, detection bias, and confounding, the observational nature of the pharmacoepidemiological design remains an important constraint in this initial report. Direct site monitoring of subject management was not conducted in this observational international study for reasons of cost and logistics, but for subsequent hypothesis-driven research this would enhance data accuracy and completeness, reduce potential bias, and improve the confidence, interpretability, and generalizability of the results obtained. Within the recognized constraints of pharmacoepidemiological methodology employed, however [47], this study supports the growing number of trials that have shown conversion to sirolimus to provide a potent, effective, and generally safe option for long-term immunosuppression in renal transplantation, particularly when introduced later in the transplant course. However, we emphasizes that its optimal use to preserve long-term functional graft survival remains to be established by formal and rigorous randomized prospective study [17, 30, 65, 66].

## Figures and Tables

**Table 1:** Demographics and baseline characteristics of subjects.

Time of conversion after transplant	Early	Late	Total
Number of subjects	187	297	484
Number of subjects by region			
(i) North America	55	84	139
(ii) South America	129	207	336
(iii) Europe	2	4	6
(iv) Other	1	2	3
Mean age (years)^∗^	45 ± 14	35 ± 15	39 ± 15
Gender (male)	120 (64%)	178 (60%)	298 (62%)
Ethnicity^∗^			
(i) Caucasian	141 (75%)	212 (71%)	353 (73%)
(ii) Black	31 (17%)	29 (10%)	60 (12%)
(iii) Hispanic	12 (6%)	46 (16%)	58 (12%)
(iv) Asian	1 (1%)	3 (1%)	4 (1%)
(v) Other	2 (1%)	7 (2%)	9 (2%)
Primary kidney disease			
(i) Glomerulonephritis	50 (27%)	102 (34%)	152 (31%)
(ii) Diabetes mellitus	17 (9%)	17 (6%)	34 (7%)
(iii) Hypertension	31 (17%)	42 (14%)	73 (15%)
(iv) Polycystic kidney disease	10 (5%)	25 (8%)	35 (7%)
Transplant number			
(i) First transplant	167 (89%)	273 (92%)	440 (91%)
(ii) Retransplant	20 (11%)	24 (8%)	44 (9%)
Donor source^∗^			
(i) Deceased donor	135 (72%)	132 (44%)	267 (55%)
(ii) Living donor	48 (26%)	162 (55%)	210 (43%)
(iii) Not reported	4 (2%)	3 (1%)	7 (2%)
Panel reactive antibodies (%)	8 ± 20	6 ± 16	7 ± 18
Time to start of sirolimus (months)	3 ± 3	61 ± 38	39 ± 41
Posttransplant followup (months)	27 ± 14	24 ± 12	25 ± 13
Reason for commencing sirolimus			
(i) Deteriorating graft function	38 (20.3%)	108 (36.4%)	146 (30.2%)
(ii) Adverse effects of prior immunosuppressants	55 (29.4%)	89 (30.0%)	144 (29.8%)
(iii) Routine practice within centre	26 (13.9%)	14 (4.7%)	40 (8.3%)
(iv) Delayed graft function	29 (15.5%)	2 (0.7%)	31 (6.4%)
(v) Rejection on prior immunosuppression	5 (2.7%)	9 (3.0%)	14 (2.9%)
(vi) Contraindications to an other immunosuppression	4 (2.1%)	1 (0.3%)	5 (1.0%)
(vii) Other	30 (16.0%)	74 (24.9%)	104 (21.5%)

*P < 0.05.

**Table 2:** Mean (and SD) doses and blood levels of sirolimus and calcineurin inhibitors in subjects receiving these medications at specified time relative to conversion to sirolimus.

	Conversion	Month 3	Month 6	Year 1	Year 2
All subjects (*n* = 484)					
Sirolimus dose (mg/d)	4.3 ± 3.8	2.9 ± 2.1	2.8 ± 1.6	2.6 ± 1.5	2.3 ± 1.1
Sirolimus level (*μ*g/L)	5.4 ± 5.5	10.1 ± 4.6	9.3 ± 4.0	9.0 ± 3.8	8.5 ± 2.9
Tacrolimus dose (mg/d)	6.0 ± 4.1	5.4 ± 3.3	5.3 ± 2.8	5.3 ± 3.6	4.5 ± 2.2
Tacrolimus level (*μ*g/L)	7.3 ± 3.6	6.7 ± 3.8	5.8 ± 3.2	5.8 ± 2.5	5.0 ± 2.2
Cyclosporine dose (mg/d)	188 ± 183	181 ± 111	154 ± 107	128 ± 74	167 ± 107
Cyclosporine level (*μ*g/L)	194 ± 181	220 ± 129	186 ± 101	158 ± 91	159 ± 91
Converted early (*n* = 187)					
Sirolimus dose (mg/d)	4.3 ± 2.9	3.2 ± 2.6	2.8 ± 1.6	2.7 ± 1.8	2.1 ± 0.7
Sirolimus level (*μ*g/L) < 1 yr	8.4 ± 5.9	10.4 ± 4.6	9.7 ± 4.0	9.0 ± 4.0	8.4 ± 3.1
Tacrolimus dose (mg/d)	7.5 ± 5.7	6.9 ± 4.4	6.4 ± 3.5	7.0 ± 4.7	5.5 ± 2.7
Tacrolimus level (*μ*g/L)	8.7 ± 4.0	7.1 ± 3.8	6.5 ± 3.8	6.1 ± 2.5	6.0 ± 3.2
Cyclosporine dose (mg/d)	283 ± 309	220 ± 148	186 ± 155	130 ± 92	NS
Cyclosporine level (*μ*g/L)	270 ± 237	204 ± 137	120 ± 60	99 ± 63	88 ± 46
Converted late (*n* = 297)					
Sirolimus dose (mg/d)	4.3 ± 4.4	2.7 ± 1.7	2.7 ± 1.6	2.6 ± 1.3	2.4 ± 1.2
Sirolimus level (*μ*g/L) > 1 yr	4.1 ± 4.8	10.5 ± 4.6	9.7 ± 4.0	9.0 ± 4.0	8.4 ± 3.1
Tacrolimus dose (mg/d)	4.7 ± 2.1	4.5 ± 2.0	4.5 ± 1.8	4.1 ± 1.9	4.0 ± 1.7
Tacrolimus level (*μ*g/L)	6.1 ± 2.6	6.3 ± 3.8	5.3 ± 2.7	5.6 ± 2.5	4.4 ± 1.0
Cyclosporine dose (mg/d)	157 ± 99	150 ± 56	140 ± 79	128 ± 72	167 ± 107
Cyclosporine level (*μ*g/L)	184 ± 172	225 ± 127	120 ± 66	99 ± 63	88 ± 46

**Table 3:** Cox analysis of time to rejection, graft loss, or death following renal transplantation.

Covariates	Time to acute rejection			Time to graft loss			Time to death		
	HR	95% CI	*P*	HR	95% CI	*P*	HR	95% CI	*P*
Conversion before or after 1 year	6.604	(2.145, 20.33)	0.0010	1.185	(0.634, 2.216)	0.5949	1.287	(0.507, 3.268)	0.5958
Recipient age (years)	0.969	(0.939, 1.000)	0.0511	0.985	(0.967, 1.004)	0.1190	1.069	(1.030, 1.110)	0.0004
Recipient race (Black versus non-Black)	1.178	(0.377, 3.686)	0.7781	1.627	(0.777, 3.403)	0.1965	0.870	(0.251, 3.022)	0.8271
Prior kidney transplant (yes versus no)	0.979	(0.213, 4.500)	0.9783	1.527	(0.611, 3.817)	0.3655	3.023	(0.783, 11.67)	0.1084
Current PRA	0.995	(0.929, 1.066)	0.8877	0.980	(0.932, 1.030)	0.4278	0.980	(0.920, 1.043)	0.5207
Anti-CD25 induction therapy versus none	3.707	(1.276, 10.77)	0.0160	1.174	(0.528, 2.607)	0.6941	1.307	(0.412, 4.144)	0.6497
ATG/OKT3 induction therapy versus none	1.363	(0.444, 4.181)	0.5880	1.118	(0.573, 2.179)	0.7438	0.696	(0.241, 2.009)	0.5033
Donor organ: cadaver versus living	1.979	(0.375, 2.553)	0.9654	1.306	(0.731, 2.333)	0.3666	2.711	(0.893, 8.235)	0.0785

**Table 4:** Principal adverse events by category and by time after transplant.

Time of conversion after transplant	Total	<1 year	>1 year
Number of subjects	458	187	297
Any event^∗^	458 (95%)	182 (97%)	276 (93%)
Vascular system	241 (50%)	103 (55%)	138 (47%)
Hypertension	226 (47%)	95 (51%)	131 (44%)
Cardiac system^∗^	46 (10%)	25 (13%)	21 (7%)
Coronary artery disease	8 (2%)	4 (2%)	4 (1%)
Myocardial infarction	6 (1%)	1 (0.5%)	5 (2%)
Metabolism and nutrition	249 (51%)	102 (55%)	147 (50%)
Hyperlipidemia^∗^	128 (26%)	66 (35%)	62 (21%)
Diabetes	67 (14%)	21 (11%)	46 (16%)
Hypercholesterolemia	31 (6%)	7 (4%)	24 (8%)
Blood and lymphatic system	146 (30%)	60 (32%)	86 (29%)
Anaemia	116 (24%)	44 (24%)	72 (24%)
Leukopenia	29 (6%)	13 (7%)	16 (5%)
Thrombocytopenia	7 (1%)	3 (2%)	4 (1%)
Gastrointestinal system	127 (26%)	41 (22%)	86 (29%)
Mouth ulceration	2 (0.5%)	0 (0%)	2 (1%)
Diarrhoea	67 (14%)	21 (11%)	46 (16%)
Infections	288 (60%)	120 (64%)	168 (57%)
Cytomegalovirus infection^∗^	57 (12%)	35 (19%)	22 (7%)
Urinary tract infection	120 (25%)	54 (29%)	66 (22%)
Musculoskeletal system	73 (15%)	25 (13%)	48 (16%)
Bone pain	3 (0.5%)	1 (0.5%)	2 (0.5%)
Renal and urinary system	108 (22%)	33 (18%)	75 (25%)
Proteinuria^∗^	49 (10%)	10 (5%)	39 (13%)
Lymphocele	9 (2%)	6 (3%)	3 (1%)
Edema	28 (6%)	16 (9%)	12 (4%)
Peripheral edema	40 (8%)	14 (7%)	26 (9%)
Respiratory system	45 (9%)	17 (9%)	28 (9%)
Pneumonitis	3 (0.5%)	0 (0%)	3 (1%)
Neoplasms (malignant)	43 (9%)	12 (6%)	31 (10%)
Skin rash	1 (0.2%)	1 (0.5%)	0 (0%)

*P ≤ 0.05.

## Abbreviations

mTOR: Mammalian target of rapamycin
GFR: Glomerular filtration rate
CNI: Calcineurin inhibitor
SRL: Sirolimus
TAC: Tacrolimus
CSA: Cyclosporine
MMF: Mycophenolate mofetil
HR: Hazard ratio
CCr: Calculated creatinine clearance
BCAR: Biopsy-confirmed acute rejection.
